# Outcomes, efficacy and risk factors of 27-Gauge vitrectomy for diabetic tractional retinal detachment in Japanese patients

**DOI:** 10.1007/s10384-024-01135-6

**Published:** 2024-11-06

**Authors:** Risa Nishigushi, Ayumi Usui-Ouchi, Yoshihito Sakanishi, Kazunori Tamaki, Keitaro Mashimo, Rei Ito, Toshiro Sakuma, Nobuyuki Ebihara, Shintaro Nakao

**Affiliations:** 1 Department of Ophthalmology, Juntendo University Urayasu Hospital, 2-1-1 Tomioka, Urayasu, Chiba 279-0021 Japan; 2 Department of Ophthalmology, St. Luke’s International Hospital, Tokyo, Japan; 3 Department of Ophthalmology, Juntendo University Graduate School of Medicine, 2-1-1 Hongo, Bunkyo-ku, Tokyo, 113-0033 Japan

**Keywords:** Proliferative diabetic retinopathy, Tractional retinal detachment, 27-gauge vitrectomy, Postoperative outcomes, Complications

## Abstract

**Purpose:**

Diabetic retinopathy leads to vision-threatening complications, such as proliferative diabetic retinopathy and tractional retinal detachment (TRD) and is a major global health concern. Despite advancements in vitrectomy techniques, challenges exist in managing postoperative complications and long-term visual acuity. This study aimed to evaluate postoperative outcomes of 27-gauge pars plana vitrectomy (27 g PPV) for diabetic TRD and identify associated risk factors.

**Study Design:**

Retrospective study.

**Methods:**

This study included 94 eyes of 74 patients who underwent 27 g PPV for diabetic TRD between July 2017 and September 2022 at Juntendo University Urayasu Hospital, Japan. Patient demographics, preoperative characteristics, intraoperative details, and postoperative outcomes were examined. Statistical analyses were performed to identify factors influencing postoperative visual acuity.

**Results:**

Mean follow-up duration was 23.1 ± 14.6 months. Postoperatively, visual acuity (LogMAR) improved significantly from 1.34 ± 0.82 to 0.65 ± 0.79 (*P* < 0.0001). Postoperative complications included persistent vitreous hemorrhage (15%) and neovascular glaucoma (4%). Final retinal reattachment rate was 97%. Preoperatively, macular detachment (*P* < 0.0001) and Grade IV TRD (*P* < 0.0001) severity were significantly associated with poor final best corrected visual acuity (*P* < 0.0001). Preoperative macular detachment (*P* < 0.0001), Grade IV TRD (*P* < 0.0001), intraoperative iatrogenic breaks (*P* = 0.031), and postoperative neovascular glaucoma (*P* < 0.0001) were identified as significant predictors of poor postoperative visual outcomes through multivariate analysis.

**Conclusion:**

This study highlights the efficacy of 27 g PPV in improving visual acuity in patients with diabetic TRD. Despite favorable outcomes, attention to preoperative risk factors and meticulous surgical techniques remain crucial for optimizing long-term visual prognosis in these patients.

## Introduction

Globally, the prevalence of diabetes mellitus has been steadily increasing, with projections indicating a continuous increase in the coming decades. Diabetic retinopathy affects approximately one-third of individuals with diabetes, 28.54 million people worldwide are estimated to have vision-threatening diabetic retinopathy; by 2045, the numbers are projected to increase up to 44.82 million [1].

Proliferative diabetic retinopathy (PDR) poses a significant threat to vision and represents an advanced stage of diabetic retinopathy, characterized by the development of retinal neovascularization. Among these complications, tractional retinal detachment (TRD) is the most severe vision-impairing complication, resulting from the contraction of fibrovascular membranes tightly adherent to the retina, necessitating vitrectomy for membrane removal and retinal reattachment. However, despite the effectiveness of vitrectomy, particularly in cases of PDR complicated with TRD, the postoperative landscape remains fraught with challenges. Complications such as neovascular glaucoma (NVG), rebleeding, and recurrent retinal detachment due to retinal proliferation can significantly affect long-term prognosis [2]. It is against this backdrop that the need for a comprehensive understanding of postoperative outcomes becomes imperative.

Japan has been notably successful in reducing the number of visually impaired individuals with diabetic retinopathy despite the yearly increase in patients with diabetes mellitus [3]. In the national survey of the fiscal year 1988, diabetic retinopathy was the primary cause of visual impairment certifications; subsequently it fell to the second place in the following two surveys. Remarkably, in the most recent survey, it ranked third, trailing behind retinitis pigmentosa [3]. This decline in visually impaired cases associated with diabetic retinopathy in Japan may be attributed to advancements in diabetes mellitus and diabetic retinopathy treatments, such as the introduction of intravitreal injection of anti-vascular endothelial growth factor (VEGF) agents, systemic administration of dipeptidyl peptidase-4 (DPP-4) inhibitors, sodium-glucose co-transporter-2 (SGLT2) inhibitors, and glucagon-like peptide-1 (GLP-1) receptor agonists, as well as improvements in vitrectomy systems [4].

The introduction of Micro Incision Vitrectomy Systems (MIVS) using 23-gauge (23 g) and 25-gauge (25 g) techniques marked a pivotal advancement, significantly enhancing the precision and outcomes of vitrectomy procedures, offering benefits such as reduced operative time, enhanced wound healing, decreased postoperative inflammation, minimized postoperative discomfort, and accelerated visual recovery compared with conventional 20-gauge pars plana vitrectomy (PPV) [5–7]. In 2010, Oshima et al. pioneered the use of an even smaller, sutureless 27-gauge (27 g) PPV system, marking a significant advancement [8]. Initially employed in cases of macular diseases and simple vitreous hemorrhage, the continuous evolution of 27 g PPV technology, including enhancements in instrument rigidity, shape, cut rate, stability of suction flow rate, and illumination systems, has expanded its utility, leading to its adoption in more complex cases such as proliferative vitreoretinopathy and diabetic TRD [9–13]. However, reports on the 27 g PPV for diabetic TRD are limited in terms of case numbers and observational periods, and the risk factors for postoperative outcomes and complications have not been thoroughly evaluated.

This study reviewed the postoperative visual and anatomical outcomes of 27 g PPV for diabetic TRD retrospectively and assessed the associated risk factors through a comprehensive examination of patient characteristics, including both systemic and ocular factors, surgical techniques, and factors influencing postoperative visual acuity in Japan, one of the countries most commonly utilizing 27 g PPV.

## Materials and methods

### Subjects and study design

This was a retrospective, consecutive cohort study performed at the Juntendo University Urayasu Hospital, Chiba, Japan. Medical records were reviewed for all patients who underwent transconjunctival 27 g PPV for TRD secondary to PDR between July 1, 2017 and September 30, 2022. Eyes with a follow-up period of less than 6 months, those with other causes of proliferative vitreoretinal disease, previously vitrectomized eyes, or those undergoing 20-, 23-, or 25-gauge vitrectomy were excluded.

**Fig. 1:**
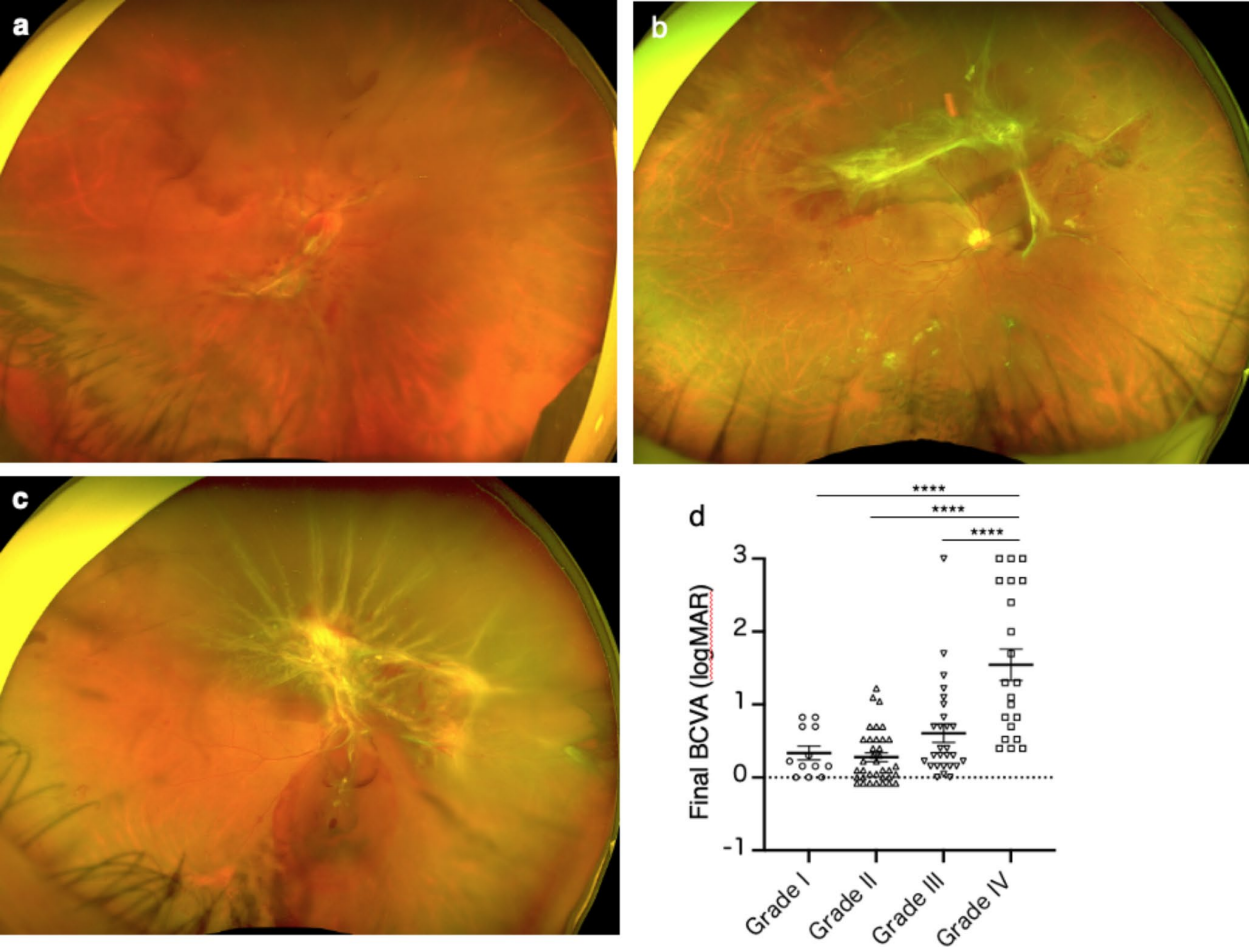
Fundus photograph images representing severity grade of diabetic tractional retinal detachment (d-TRD) and visual outcome of final best corrected visual acuity (BCVA) in each grade (a) Grade I d-TRD, (b) Grade II d-TRD, and (c) Grade III d-TRD. (d) Preoperative grade IV d-TRD is significantly associated with poor final BCVA. One-way ANOVA Tukey’s test; ****P < 0.0001

Patient systemic background data, including age, sex, hemoglobin A1c (HbA1c), fasting blood sugar, presence of overt proteinuria, serum creatinine, stage of chronic kidney disease (CKD), serum albumin, and blood pressure, were obtained. Preoperative findings were recorded, including best corrected visual acuity (BCVA; logMAR), presence of panretinal photocoagulation (PRP), presence of combined tractional and rhegmatogenous detachment (CTRRD), macular detachment, severity of TRD (Grade I–IV), preoperative anti-VEGF medication, macular traction, and epiretinal membrane (ERM). The severity of TRD was graded according to previous literature [14] as follows (Fig. 1a-c): Grade I, multiple-point adhesion with or without one-site plaque-like broad adhesion; Grade II, more than one broad adhesion and fewer than three sites located posterior to the equator; Grade III: more than three broad adhesion sites located posterior to the equator or extending beyond the equator within one quadrant; and Grade IV: broad adhesions extending beyond the equator in more than one quadrant.

Intraoperative findings included operating time, presence of internal limiting membrane (ILM) peeling, iatrogenic break, simultaneous cataract surgery, gas tamponade, silicone oil (SO) tamponade, and scleral wound suture.

Postoperative findings included BCVA (logMAR), intraocular pressure (IOP) on the day after surgery, presence of ERM, persistent vitreous hemorrhage (VH), recurrent retinal detachment, NVG, and SO removal. Persistent vitreous hemorrhage was defined as a hemorrhage in which the fundus is invisible at least one week after surgery and reoperation was considered. We evaluated the association between these factors and final BCVA.

### Surgical techniques

All vitrectomies were performed under local sub-tenon anesthesia by one of six skilled and experienced surgeons (AU, YS, KT, KM, RI, and TS) using the Constellation Vitrectomy 27 + Total Plus Pak vitrectomy system (Alcon Laboratories) and 27G chandelier illumination fiber (Oshima vivid, Synergetics). Cannulas were created by conjunctival displacement and oblique-angled sclerotomy. Preoperative intravitreal anti-VEGF injections were administered in cases where each surgeon determined the necessity based on the activity of neovascularizations. Before vitrectomy, phacoemulsification and intraocular lens implantation with a 2.4 mm corneal incision were performed using the same machine for eyes with cataracts. A wide-angle non-contact viewing system (Resight^®^; Carl Zeiss Meditec AG) was used and core vitrectomy was performed in all cases using a cut rate of 7500–20,000 cuts per minute (cpm) and linear aspiration of 0–650 mmHg. The areas of fibrovascular proliferation were segmented and dissected using a 27-g beveled cutter probe, as needed, assisted by ILM forceps and 27-g scissors. ILM removal was performed when traction by the proliferating membrane extended close to the macula and the surgeon considered it necessary. After all fibrovascular proliferations were removed from the retina, pan-photocoagulation was performed using an endophotocoagulation probe. Fluid-air exchange, fluid-gas exchange (20% sulfur hexafluoride; SF6), or SO tamponade were performed as needed. In cases of tractional retinal detachment involving the macula without retinal breaks, and in cases with retinal breaks and limited retinal detachment, gas tamponade was performed. In cases with retinal breaks and extensive retinal detachment, or when the vitreous removal was deemed incomplete, SO tamponade was used. SO was injected through a 27-gauge trocar without port enlargement at 30 mmHg pressure. All sclerotomy sites were inspected after removal, or the cannulas and sclerotomy sites were sutured with 7 − 0 vicryl, whenever leakage was observed. The SO was removed–3–6 months after initial vitrectomy.

### Statistical analysis

Statistical analysis between preoperative logMAR BCVA and final logMAR BCVA was performed using the Wilcoxon signed-rank test, and the statistical interaction between preoperative TRD severity and final logMAR BCVA was analyzed using one-way analysis of variance with Tukey’s test. Each of the pre-, intra-, and post-operative data were divided into groups as “presence” and “absence.” Factors associated with the final BCVA were statistically analyzed using univariable and multivariable analyses. Univariable analyses were performed using the Mann–Whitney U test. Subsequently, we conducted multiple regression analysis (stepwise method) to determine the significant factors affecting the final logMAR as the dependent variable, with the presence (1) or absence (0) of each variable that significantly influenced the final logMAR as independent variables. IBM SPSS version 28 (SPSS Inc.) was used for statistical analysis.

### Ethical considerations

This study adhered to the tenets of the Declaration of Helsinki and was approved by the Institutional Review Board of Juntendo Urayasu Hospital (approval number: E22-0234). Informed consent was obtained from the patients involved in this study.

## Results

Ninety-four eyes from a cohort of 74 consecutive patients diagnosed with PDR with TRD were included in this study. The mean observation period was 23.1 ± 14.6 months.

The baseline demographic characteristics of the study cohort, comprising 94 eyes from 74 patients undergoing 27 g MIVS for PDR with TRD, were thoroughly examined (Table 1). The majority of participants were men (64%), with a mean age of 51.0 ± 12.9 years. Metabolic parameters, including HbA1c (8.0 ± 2.1%) and fasting blood glucose (141.5 ± 41.2 mg/dl), were within the expected range for patients with diabetes. Notably, 76% of patients exhibited overt proteinuria. Evaluation of the CKD stages revealed a normal distribution up to Stage 5, with the most prevalent being Stage 2 (41%). Systolic and diastolic blood pressure values were measured at 132.5 ± 22.9 mmHg and 81.1 ± 13.6 mmHg, respectively. No systemic factors were significantly associated with the final BCVA or the risk of postoperative complications (data not shown).

**Table 1:** Demographic characteristics and clinical findings

Characteristics & findings	*n* = 96
Systemic characteristic	
Age (y)	51.0 ± 12.9 (27–77)
Male/female (%)	47 (63.5)/27 (36.5)
HbA1c (%)	8.0 ± 2.1(5.2–14)
Fasting blood sugar (mg/dl)	141.5 ± 41.2 (77–234)
Overt proteinuria (%)	56 (76)
CKD stage (%)	
Normal-stage 1	18 (24)
Stage 2	29 (41)
Stage 3	19 (26)
Stage 4 & 5	8 (11)
Serum creatinine (mg/dl)	1.12 ± 0.96 (0.17–6.97)
Serum albumin (g/dl)	3.86 ± 0.64 (2–4.7)
Systolic blood pressure (mmHg)	132.5 ± 22.9 (89–195)
Diastolic blood pressure (mmHg)	81.1 ± 13.6 (61–141)
Preoperative findings	
BCVA (logMAR)	1.34 ± 0.82
Panretinal photocoagulation (%)	66 (69)
CTRRD (%)	30 (31)
Macular detachment (%)	39 (40)
Severity of TRD (%)	
Grade I	12 (13)
Grade II	36 (37)
Grade III	27 (28)
Grade IV	21 (11)
Preoperative anti-VEGF medication (%)	33 (34)
Macular traction (%)	66 (69)
Intraoperative findings	115 ± 69
Operating time (min)	56 (58)
Simultaneous cataract surgery(%)	53 (55)
ILM peeling (%)	37 (39)
Iatrogenic break (%)	
Gas tamponade (%)	51 (53)
Silicone oil tamponade (%)	18 (19)
Scleral wound suture (%)	2 (2.6)

CKD: chronic kidney disease, BCVA: best corrected visual acuity, CTRRD: combined tractional and rhegmatogenous detachment, TRD: tractional retinal detachment ILM: internal limiting membrane, VEGF, vascular endothelial growth factor

Regarding preoperative findings (Table 1), the BCVA logMAR was 1.34 ± 0.82. PRP was observed in 69% of the eyes, while 31% exhibited CTRRD. Macular detachment and traction were observed in 40% and 69% of the eyes, respectively. The severity of TRD varied Grade II (37.5%) being the most prevalent. Preoperative anti-VEGF medication was administered to 34% of the eyes.

Several intraoperative factors such as ILM removal, occurrence of iatrogenic breaks, simultaneous cataract surgery, and utilization of gas and SO were assessed. The average operative time was 115 ± 69 min (Table 1).

Postoperatively, improvements were noted in visual acuity, with a significant reduction in mean logMAR BCVA from 1.34 ± 0.82 to 0.65 ± 0.79 (*P* < 0.0001) (Fig. 2). Remarkably, 86% of the patients achieved an improvement or maintenance of visual acuity of 0.2 logMAR or better.

Postoperative complications included persistent vitreous hemorrhage (15%), transient low intraocular pressure (2%), postoperative ERM (5%), recurrent retinal detachment (7%), and NVG (4%) (Table 2). The final retinal reattachment rate was 97%, considering that the SO was not removed because of recurrent retinal detachment in three eyes.

**Table 2:** Postoperative complications

Complications	*n* = 96 (%)
Persistent vitreous hemorrhage	15 (16)
Epiretinal membrane	14 (15)
Recurrent retinal detachment	7 (7)
Neovascular glaucoma	4 (4)
Transient hypotony	2 (2)

The impact of various factors on postoperative visual acuity was also explored. Grade IV patients exhibited significantly poorer final visual acuity than patients with any other grade of TRD (*P* < 0.0001) (Fig. 1d). Preoperative factors, including macular detachment and Grade IV, as well as intraoperative factors, such as the occurrence of iatrogenic breaks and ILM peeling, significantly affected postoperative visual acuity (*P* < 0.0001) (Table 3). Multivariate analysis identified preoperative macular detachment (*P* < 0.0001), preoperative Grade IV (*P* < 0.0001), intraoperative iatrogenic break (*P* = 0.031), and postoperative NVG (*P* < 0.0001) as significant predictors of poor postoperative vision (Table 4).

**Table 3:** Univariate analysis of risk factors for final BCVA logMAR

Clinical features	Postoperative VA		*P* value
	Presence	Absence	
Preoperative feature			
Macular detachment	1.12 ± 0.83	0.33 ± 0.58	< 0.0001
Transient hypotony	0.74 ± 0.70	0.61 ± 0.84	NS
Anti-VEGF injection	0.63 ± 0.81	0.70 ± 0.78	NS
Grade IV TRD	1.55 ± 0.99	0.40 ± 0.50	< 0.0001
Macular traction	0.79 ± 0.84	0.36 ± 0.59	0.0030
CTRRD	1.11 ± 0.99	0.45 ± 0.59	0.0005
Intraoperative feature			
Iatrogenic break	0.99 ± 0.91	0.44 ± 0.63	0.0003
ILM peeling	0.87 ± 0.84	0.40 ± 0.67	< 0.0001
Postoperative feature			
Persistent vitreous hemorrhage	0.59 ± 0.77	0.67 ± 0.80	NS
Postoperative ERM	0.62 ± 1.02	0.66 ± 0.75	NS
Recurrent retinal detachment	1.95 ± 1.06	0.55 ± 0.68	0.0002
Neovascular glaucoma	1.78 ± 1.42	0.61 ± 0.73	0.0429

*BCVA*: best corrected visual acuity, *TRD*: tractional retinal detachment, *CTRRD*: combined tractional and rhegmatogenous detachment, *ILM*: internal limiting membrane, *ERM*: epiretinal membrane, NS: not significant

**Table 4:** Multivariate analysis for risk factors for final BCVA logMAR

Clinical feature	β	*P* value	95%
Preoperative macular detachment	0.312	< 0.001	0.228–0.775
Preoperative grade IV TRD	0.328	< 0.001	0.269–0.984
Intraoperative iatrogenic break	0.184	0.031	0.028–0.568
Postoperative NVG	0.302	< 0.001	0.581–1.805

*BCVA*, best corrected visual acuity; *TRD*, tractional retinal detachment; *NVG*, neovascular glaucoma

## Discussion

This study shows that 27 g MIVS is effective even in complex and severe cases such as PDR complicated by TRD. The mean postoperative logMAR visual acuity significantly improved from 1.34 to 0.65, with 86% of patients achieving an improvement or maintenance of visual acuity of 0.2 logMAR or better. These outcomes underscore the efficacy of the 27 g MIVS in enhancing visual acuity in patients with diabetic TRD. Comparing our results with literature, our findings align with previous studies demonstrating the efficacy of 27 g MIVS. Cruz-Iñigo & Berrocal (2017) Khan et al. (2018), and Chen et al. (2021) published the surgical results of 27 g MIVS for PDR with TRD (observation period > 6 months), with comparable results for postoperative visual acuity and postoperative complications [14–16] (Table 5). To the best of our knowledge, this study includes the largest number of cases to date, which enhances the reliability of the findings.

**Table 5:** Comparison of different studies with 27-gauge PPV in diabetic TRD

Study (publication year)	Number of eyes	Mean age (y)	Postoperative BCVA	Postoperative complication (%)
Chen et al. (2021) [13]	21	56.4 ± 8.5	0.79 ± 0.55	8 (38)
Cruz-Iñigo & Berrocal (2017) [14]	12	53.0 ± 7.0	0.92 ± 0.40	3 (25)
Khan et al. (2018) [15]	49	NA	0.78 ± 0.72	NA
Present study	96	51.0 ± 12.9	0.65 ± 0.8	23 (24)

*PPV*: pars plana vitrectomy, *TRD*: tractional retinal detachment, BCVA: best corrected visual acuity

The advantages of 27 g MIVS over 23 g and 25 g MIVS include better wound closure and fewer wound closure-related complications [15, 17]. In this report, scleral sutures were performed only in two cases other than the SO cases, and only two cases had a low IOP the day after surgery. The absence of scleral sutures may have been associated with reduced postoperative discomfort. In addition, no case of infectious endophthalmitis occurred.

The 27 g MIVS is reported to deliver comparable surgical outcomes to the 25 g MIVS for Diabetic TRD [14], owing to enhanced rigidity of the instrument, and the high cut rate of the cutter. Furthermore, closer distance between the retinal surface and suction opening due to the bevel design allows the cutter to be inserted into the narrow space under fibroproliferative tissue, which may be useful in the operation of diabetic TRD.

A meta-analysis of 36 studies (3720 eyes) over 22 years regarding vitrectomy for diabetic TRD with 20-, 23-, 25-, and 27-g systems, reported in 2023 that the factors affecting retinal reattachment were preoperative anti-VEGF medication and MIVS, and the factor affecting final logMAR was preoperative visual acuity (logMAR) [18]. Compared with our report, postoperative complications were similar to those in the previous report, and final visual acuity was better (final BCVA (logMAR): 0.65 vs. 0.94, postoperative VH: 16 vs. 22.5%, postoperative NVG: 4 vs. 5.3%). In this report, the factors affecting the final postoperative visual acuity were preoperative macular detachment, preoperative Grade IV TRD, iatrogenic breaks, and postoperative NVG, suggesting the need for careful attention to the previously described risks even in cases with 27 g MIVS.

The identification of Grade IV as a significant factor influencing poor final visual acuity emphasizes the importance of grading systems in prognostic outcomes. Understanding the severity of TRD preoperatively can guide clinicians in setting realistic expectations for visual acuity improvement. Surgeons must exercise extreme care when processing the retina in traction owing to proliferative tissue to avoid iatrogenic breaks.

Despite its strengths, this study has several limitations. The retrospective design introduced the possibility of selection bias, and the reliance on medical records may have resulted in incomplete data. Additionally, the study was conducted at a single institution, which limits the generalizability of the findings. Furthermore, the observational nature of this study precludes the establishment of causation.

This extensive analysis of a large sample size of 27 g PPV in patients with diabetic TRD underscores the benefits and safety of this system in treating diabetic TRD, potentially leading to a reduction in severe visual impairment due to diabetic retinopathy. However, it remains imperative to meticulously address severe TRD, macular detachment, iatrogenic breaks, and postoperative NVG to optimize the long-term visual outcomes in patients with diabetic TRD.

## Data Availability

Data presented in this study are available upon reasonable request from the corresponding author. The data are not publicly available due to privacy concerns.
